# Supplementary material for: Empirical methods for controlling false positives and estimating confidence in ChIP-Seq peaks
Source: BMC Bioinformatics. 2008 Dec 5;9:523. doi: 10.1186/1471-2105-9-523 (PMC2628906; doi:10.1186/1471-2105-9-523)
Supplement: Additional File 1 — A variety of html documents from the USeq web site detailing the available applications, their best usage, output file type descriptions, command line menus, etc. [file 1471-2105-9-523-S1.zip › USeqUserGuides/JavaDoc.zip!/JavaDoc/util/bio/annotation/class-use/ScoredCoordinate.html]

Uses of Class util.bio.annotation.ScoredCoordinate


|  |  |  |
| --- | --- | --- |
| **Overview** \| **Package** \| **Class** \| **Use** \| **Tree** \| **Deprecated** \| **Index** \| **Help** | |  |
| PREV   NEXT | **FRAMES**    **NO FRAMES**     **All Classes** |  |


---


## **Uses of Class util.bio.annotation.ScoredCoordinate**

| Packages that use ScoredCoordinate | |
| --- | --- |
| **util.bio.annotation** | For representing gene annotation. |

Uses of ScoredCoordinate in util.bio.annotation

| Methods in util.bio.annotation that return ScoredCoordinate | |
| --- | --- |
| `static ScoredCoordinate[]` | `ScoredCoordinate.makeRanked(Coordinate[] c)`             Returns an array of ScoredCoordinate where the score is the index position of the Coordinate[]. |

| Methods in util.bio.annotation with parameters of type ScoredCoordinate | |
| --- | --- |
| `static java.util.HashMap` | `ScoredCoordinate.splitByChromosome(ScoredCoordinate[] sortedCoordinates)`             Split by chromosome into a HashMap of chromosome:ScoredCoordinate[]. |

---


|  |  |  |
| --- | --- | --- |
| **Overview** \| **Package** \| **Class** \| **Use** \| **Tree** \| **Deprecated** \| **Index** \| **Help** | |  |
| PREV   NEXT | **FRAMES**    **NO FRAMES**     **All Classes** |  |


---
